# Short-Term Effect of Different Taping Methods on Local Skin Temperature in Healthy Adults

**DOI:** 10.3389/fphys.2020.00488

**Published:** 2020-05-20

**Authors:** Kun Liu, Zhouying Duan, Lihua Chen, Zixing Wen, Shengqun Zhu, Qiang Qu, Wenhua Chen, Shuxin Zhang, Bo Yu

**Affiliations:** ^1^Department of Rehabilitation, School of International Medical Technology, Shanghai Sanda University, Shanghai, China; ^2^Department of Rehabilitation, Shanghai General Hospital, Shanghai Jiao Tong University, Shanghai, China; ^3^Department of Rehabilitation, Shanghai Fifth Rehabilitation Hospital, Shanghai, China

**Keywords:** kinesio tape, skin temperature, infrared thermal imaging, taping shapes and materials, erector spinae

## Abstract

**Background:**

There were limited studies on the effect of skin temperature and local blood flow using kinesio tape (KT) adhered to the skin in different taping methods. This study aimed to determine the short-term effect of KT and athletic tape (AT) on skin temperature in the lower back and explore the possible effect of different taping methods (Y-strip and fan-strip taping) on local microcirculation.

**Materials and Methods:**

Twenty-six healthy participants completed the test-retest reliability measurement of the infrared thermography (IRT), intraclass correlation coefficient (ICC), and standard error of measurement (SEM) were calculated to evaluate the reliability. Then, 21 healthy participants received different taping condition randomly for 5 times, including Y-strip of kinesio taping (KY), fan-strip of kinesio taping (Kfan), Y-strip of athletic taping (AY), fan-strip of athletic taping (Afan), and no taping (NT). Above taping methods were applied to the participants’ erector spinae muscles on the same side. Skin temperature of range of interest (ROI) was measured in the taping area through IRT at pre taping and 10 min after taping. Additionally, participants completed self-perceived temperature evaluation for different taping methods through visual analog scaling. One-way repeated-measured analysis of variance was used to compare the temperature difference among different taping methods. Bonferroni test was used for post hoc analysis.

**Results:**

There was a good test-retest reliability (ICC = 0.82, 95% CI = 0.60–0.92; SEM = 0.33; and MD = 0.91) of the IRT. Significant differences were observed in the short-term effect on skin temperature among all different taping methods (*p* = 0.012, *F* = 3.435, and η_p_^2^ = 0.147), *post hoc* test showed a higher significantly skin temperature difference in Kfan taping compared to no taping (*p* = 0.026, 95% CI = 0.051–1.206); However, no significant differences were observed among self-perceived temperature (*p* = 0.055, *F* = 2.428, and η_p_^2^ = 0.108).

**Conclusion:**

This study showed that the fan-strip of KT increased significantly the skin temperature of the waist after taping for 10 min. The application of KT may modify the skin temperature of the human body and promote local microcirculation, although it remained unclear for the real application.

## Introduction

Kinesio tape (KT) is an elastic adhesive skin tape, which has been reported to increase local blood and lymphatic flow, promote proprioceptive input, enhance muscle strength, suppress pain, improve stability, and improve range of motion (Ekiz et al., 2015; Stryker et al., 2016; Wozniak-Czekierda et al., 2017; Bischoff et al., 2018; Kasawara et al., 2018; Gulpinar et al., 2019). It differs from traditional athletic tape (AT) which is an inelastic adhesive skin tape that functions to provide fixed protection (Kase et al., 2003; De Ridder et al., 2015). In clinical application, KT is generally cut into I-strip, O-strip, X-strip, Y-strip, or fan-strip for different functions according to the characteristics of human body joint and muscle shapes. Among with them, I-strip, and O-strip were used commonly to provide stability for muscles and fascia according to the anatomical structures, X-strip was used to relieve pain owing to the effect of lifting skin, Y-strip, and fan-strip were used commonly to eliminate swelling and promote lymphatic circumfluence because of the tails (Kumbrink, 2012; Chen and Yu, 2017; Vercelli et al., 2017). However, the quantitative and reliable taping standard for reducing swelling has not been established in previous studies.

Currently, there was limited research exploring the effect of KT on local circulation and swelling relief (Kalron and Bar-Sela, 2013). Additionally, the related mechanism of KT reported in these studies are contradictory. Vercelli et al. (2017) reported that KT improved blood and lymphatic reflux through the formation of skin wrinkles which increased the gap between the skin and the underlying connective tissue, which is useful for the treatment of lymphedema, venous insufficiency, swelling, and superficial hematoma. However, another study reported that KT could promote lymphatic reflux and reduce swelling due to pressurized bandaging effect (Aguilar-Ferrandiz et al., 2014).

Numerous literatures have indicated that the local microcirculation was related closely with skin temperature (Tauchmannova et al., 1993; Ng, 2009; Gomes Moreira et al., 2017). Changes in blood flow reflected from vasoconstriction and vasodilation can result in fluctuations in skin temperature, and vice versa (Taylor et al., 1984; Pergola et al., 1993). More recently, studies have focused on the effect of KT in terms of skin temperature changes in order to seek the evidence of KT promoting circulation from the perspective of skin temperature. Slomka et al. (2018) reported that the skin temperature from infrared thermography (IRT) decreased immediately on removal and then increased significantly after I-strip taping continuously for 4 days. Similarly, Windisch et al. (2017) found that the skin temperature of the wound site in fan-strip group was higher significantly than in control group after the total knee replacement. However, inconsistent with above research, Yang and Lee (2018) indicated that the skin temperature decreased immediately after I-strip taping in the region of interest (ROI) through IRT. Therefore, there was of great contradictory in the research which explored the effect of KT on local skin temperature and related mechanism was required to be verified. In addition, although IRT was used to measure skin temperature in these studies, they did not report instrument-related test-retest reliability.

Infrared thermography which analyzes the changes and distribution of skin temperature by detecting infrared radiation emitted from the body, has been proved to be a safe, convenient, non-invasive, and painless detection method for body temperature. It plays an important role of detecting whether the surface temperature changes symmetrically (Jones et al., 2005). Therefore, it has been used widely in skin temperature changes before and after physical therapy interventions (Sefton et al., 2010; Holey et al., 2011; Roy et al., 2013; Magalhães et al., 2015).

Therefore, this study aimed to investigate the test-retest reliability of infrared thermal imaging measurement and the effect of different taping methods on local skin temperature through IRT. We hypothesized that the skin temperature would increase after fan-strip and Y strip of kinesio taping.

## Materials and Methods

### Participants

Considering a power of 0.80, a level of 0.05 in repeated measures analysis of variance, a minimum of 20 participants would be required. Finally, a total of 21 participants were recruited from local university. They were included if they: (1) Were healthy adult college students; (2) Had no history of spinal musculoskeletal (including sacroiliac) or neurosurgical injuries within the past 3 months; (3) Had not received medication in the past 3 months; (4) Had no knowledge of KT; and (5) Had intact skin over the back region and a bilateral skin temperature difference of <0.5°C prior to taping. Participants who were less than 18 years old, had a BMI > 24, had a tattoo or scar located on the back, had sustained a back injury 3 months prior to data collection, had lumbago symptoms or lumbar or sacroiliac joint lesions, were menstruating, had allergy to KT, and declined to provide a signed informed consent were excluded. The baseline characteristics of the participants are shown in Table 1.

**TABLE 1:** Participants’ characteristics ($\left[\bar{x}\right]$ ± *s*).

N	Male/Female	Age/years	Height/cm	Weight/kg	BMI/kg m^–2^
21	11/10	20.79 ± 1.58	170.72 ± 8.21	66.73 ± 12.27	22.81 ± 3.17

All participants were instructed to read and sign the inform consent. This study was approved by the Ethics committee of Shanghai University of Sport. (no. 2018071).

### Experimental Protocol

#### Laboratory Preparation

In an independent confined space of 18 square meters without any light and electronic or metal devices, an ultrafine fiber black cloth was used as a background for the whole room to absorb and reduce thermal infrared reflection. Only the researcher and participant were permitted to enter. The indoor temperature was adjusted to 24.5 ± 0.5°C, The indoor humidity was controlled at 40% (Sillero Quintana et al., 2015; Slomka et al., 2018). In addition, we also monitored the indoor atmospheric pressure and body temperature changes of the participants.

#### Participant Preparation

Participants were required to (1) refrain from any physical activity, cigarette smoking, drinking coffee, alcoholic beverages, or other stimulants for at least 24 h prior to the measurements; (2) avoid applying creams, gels, cosmetics, deodorants, or antiperspirants on the skin of the lower back region; (3) avoid direct sunlight, ultraviolet radiation, or thermal radiation prior to measurement; (4) avoid any treatment (including medication), any forms of massage, taking a shower, or bath before measurement; and (5) maintain their usual work, rest, and eating habits (Sillero Quintana et al., 2015).

#### Reliability Measurement

To investigate the reliability of IRT, a total of 28 participants were recruited to take part in the skin temperature measurement of the IRT, and finally 26 participants completed it. In the process, all participants received two temperature measurements without any intervention at the same time on day 1 and day 8, respectively. The location of measurement was consistent with ROI which was mentioned below. The inter-rater reliability was determined by correlation coefficient (ICC) to represent the relative measurement of reliability, and the standard error of measurement (SEM) of the two measurement of skin temperature was to represent the absolute measurement of reliability (Weir, 2005). The SEM and minimal difference (MD) calculation formula are as follows:

$$\begin{array}{c} \text{SEM}=\text{SD}\,d/\sqrt{2}\\ \text{MD}=\text{SEM}\times 1.96\times \sqrt{2} \end{array}$$

The SDd means the standard deviation of the difference in the two measurement of skin temperature.

#### Taping Procedures

**FIGURE 1:**
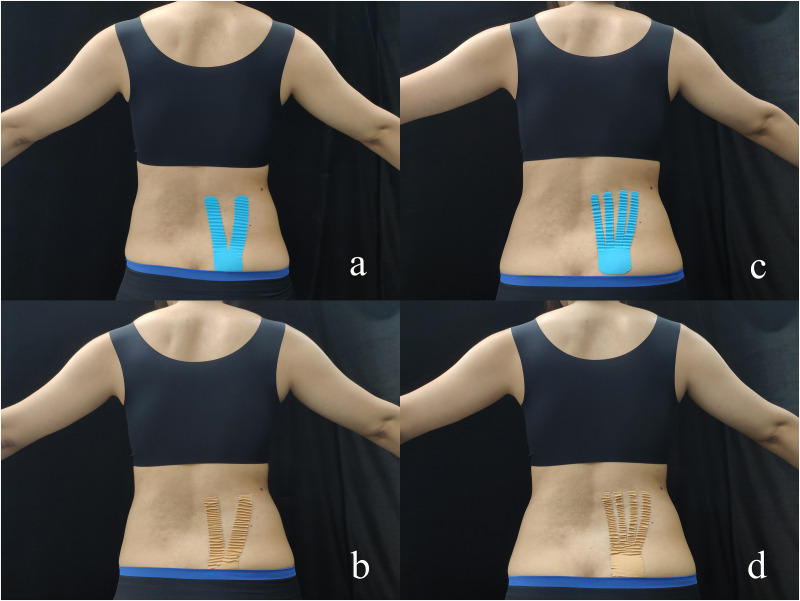
Different taping methods with Kinesio Tape and Athletic Tape. **(a)** Y-strip of kinesio taping; **(b)** Y-strip of athletic taping; **(c)** fan-strip of kinesio taping; and **(d)** fan-strip of athletic taping.

Kinesio Tex Gold (5 cm × 5 m, United States; Matheus et al., 2017) and AT with the same specification were used in this study. Prior to taping, the taping area should be removed hair, and wiped with alcohol. Each participant then received five different taping conditions, including no taping (NT), Y-strip of Kinesio Taping (KY), fan-strip of Kinesio Taping (Kfan), Y-strip of Athletic Taping (AY), and fan-strip of Athletic Taping (Afan; Figure 1). The tape was applied by a KT-certified therapist who was not informed of the purpose of the experiment and the participants were unaware about the effect of taping. Owing to the repeated-measures design, incomplete counterbalanced method was used for the randomization of taping order. Two balanced Latin Square was constructed. In the first Latin Square, the first row followed the formula 1, 2, *n*, 3, n-1, 4, n-2…, where *n* was the serial number of tapping conditions. For subsequent rows, added one to the value corresponding to the previous line, returning to 1 after *n*. The second Latin Square was a mirror image of the first Latin Square (Supplementary Material 1). This randomization method ensured to pick out any carryover effects during the statistical analysis. A week of wash-out phase between each taping conditions was to limit any learning effect. For example, the participant received first random taping condition at Monday, then second random taping condition would be performed in next Monday, and so on. According to the proportional distribution law of KT tension, Y-strip is divided equally into two tails, and the fan-strip is divided equally into four tails (Yu et al., 2016). When applying the taping, the participants stood in an anatomical position, bending forward, kept their legs straight, touched the back of their feet with their middle finger to extend their lower back, the posterior superior iliac spine was used as the location to anchor the tape, and KT was extended up to the right L1–L5 erector spinal region. According to the KT user manual, the tension of tape was approximately 10–15% (Kase et al., 2003; Figure 1). Except from different cutting shapes, the length and coverage area of different taping methods are ensured to be consistent.

#### IRT Measurement

A Spectrum 9000 MB series digital infrared thermal imaging system (United Integrated Services Co, Ltd, New Taipei, Taiwan, China) was used for measurement and imaging. IASNET2 software was used for infrared measurement and analysis. The spectral sensitivity was 7–14 μm, the minimum analytical temperature difference was 0.05 ∼ 0.08°C, the accuracy of reading value was <0.3°C, the stability of reading value was <0.3°C, the accuracy of this system was 0.1°C, and the temperature measurement range was between 10°C and 40°C (Supplementary Material 2). Infrared sensors (IRSensor Type) were used for the microbolometer, the optimal resolution was 1024 × 768, and the skin emissivity assumption was 0.98.

Before measurement, participant was instructed to sit quietly, with their lower back exposed for 15 min to adapt to the room environment and achieve thermal balance (Fernández-Cuevas et al., 2015; Moreira et al., 2017). During measurement, participant stood with his/her back to the infrared thermal meter in the anatomical position, standing 1.5 meters in front of the infrared camera with arms and hands outstretched. The camera height was adjusted to be perpendicular to the ROI to avoid parallax. It has been reported that the skin blood flow would react and tend to be stable within 10 min after application of KT (Kase and Hashimoto, 1998). Therefore, the skin temperature change was measured at prior to taping (T_0_) and 10 min after taping (T_1_) through infrared thermal imaging. During the 10-min application, the participants stood with their back against the infrared thermal camera. Measurements were performed at the time of each taping between 10:00 am and 12:00 am.

#### Selection of ROI

**FIGURE 2:**
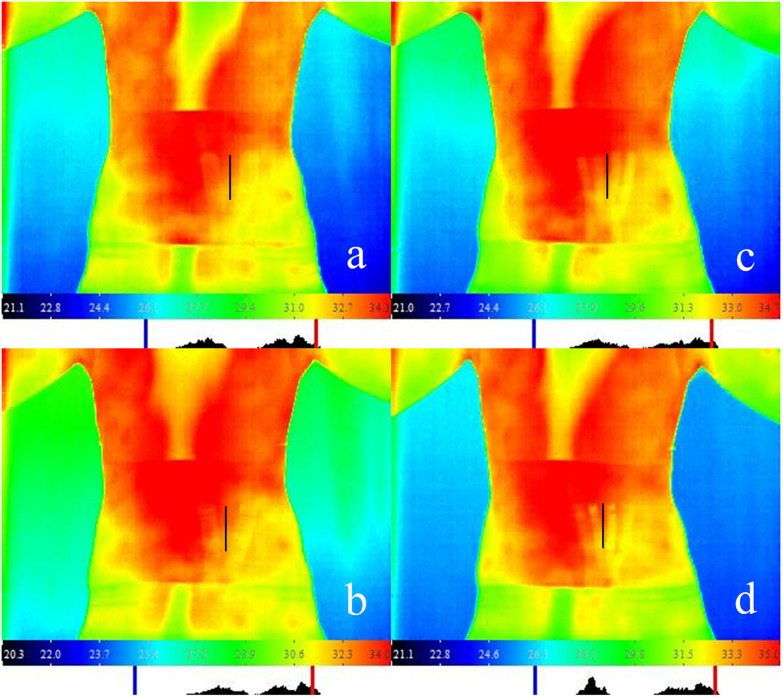
IRT measurement scenario for different taping methods with Kinesio Tape and Athletic Tape. **(a)** Y-strip of kinesio taping; **(b)** Y-strip of athletic taping; **(c)** fan-strip of kinesio taping; and **(d)** fan-strip of athletic taping. Note: In the infrared thermal image, the black line areas represent the region of interest (ROI).

Region of interest located at the site of erector spinae which not covered by taping, distanced vertically 5–6 cm away from axis of spine, and paralleled to the axis. In order to ensure that the ROI of each taping condition was consistent, since the width of tape were 5 cm, and Y-strip tape was cut equally into two tails with 2.5 cm width in each tail while fan-strip was cut equally into four tails with 1.25 cm width in each tail. The end point of the tail closest to the spine is about 1.5 cm vertically from the spine and the tails were taped in equal distribution. In this way, the width of the exposed skin area between the end point of each tail of Y-strip is about 3 cm, while the width of the exposed skin area between the end point of each tail of fan-strip is about 1 cm. Therefore, the exposed skin area ROI between the 2–3th end point of the fan-strip could coincide with the exposed skin area ROI between the two end points of the Y-strip, ROI of both were about 5–6 cm away from the axis of spine (Figure 2).

#### Self-Perceived Temperature

Finally, participants were required to describe the degree of self-perceived temperature change of the waist after 10 min for various methods of taping, and complete the visual analog scaling. 5 points means “perceived temperature has no change,” 0 points means “significant decrease in self-perceived temperature,” and 10 points means “significant increase in self-perceived temperature.”

### Statistical Analysis

The quantitative data conformed to normal distribution and was presented as mean ± standard deviation ($\left[\bar{x}\right]$ ± *s*). ICC was interpreted as follows: ICC values range > 0.9 excellent reliability, 0.75–0.9 good reliability, 0.5–0.75 moderate reliability, and <0.5 poor reliability. One-way repeated-measured analysis of variance was used to determine whether there was a significant difference among parameters of KY, Kfan, AY, Afan, and NT on the waist short-term skin temperature. Bonferroni test was used for *post hoc* analysis, the significance level was set at *p* < 0.05. Moreover, 95% confidence interval (CI) was determined, and the effect size was expressed as η_p_^2^. Small effect with 0.01 ≤ η_p_^2^ < 0.06, moderate effect with 0.06 ≤ η_p_^2^ < 0.14, and large effect with η_p_^2^ ≥ 0.14. All statistics were performed with IBM SPSS software (Version 19.0, Chicago, IL, United States).

## Results

Table 2 shows the mean value and SD*d* of the test-retest data for skin temperature measurements in the ROI with the infrared thermal imaging system. The inter-rater agreement for skin temperature measurement in ROI was good (ICC = 0.82, 95% CI = 0.60–0.92; SEM = 0.33; and MD = 0.91). (Table 2).

**TABLE 2:** Test-retest reliability of the infrared thermography measurement (*n* = 26).

T_1_	T_2_	SD*d*	ICC (95% CI)	SEM	MD	*P* value
32.82 ± 0.59	32.77 ± 0.62	0.47	0.82(0.60,0.92)	0.33	0.91	0.594

*T_1_, first temperature; T_2_, second temperature; SD*d*, standard deviation of the difference in the two measurement of skin temperature; ICC, intraclass correlation coefficient; CI, confidence interval; SEM, standard error of measurement; and MD, minimal differences.*

There was no significant difference among the room temperature (*p* = 0.329, *F* = 1.152, and η_p_^2^ = 0.054), humidity (*p* = 0.453, *F* = 0.837, and η_p_^2^ = 0.040), and air pressure of the laboratory (*p* = 0.142, *F* = 2.060, and η_p_^2^ = 0.093), and participants’ body temperature (*p* = 0.891, *F* = 0.143, and η_p_^2^ = 0.007) for different taping methods (Table 3).

**TABLE 3:** Room temperature, humidity, atmosphere of participants’ body temperature ($\left[\bar{x}\right]$ ± *s*).

	KY	Kfan	AY	Afan	NT	*F* value	*P* value	η_p_^2^
RT/°C	24.62 ± 0.62	24.95 ± 0.63	24.69 ± 0.66	24.90 ± 0.62	24.76 ± 0.64	1.152	0.329	0.054
H/%	42.04 ± 3.58	40.57 ± 2.04	41.24 ± 3.75	40.67 ± 2.18	40.90 ± 3.49	0.837	0.453	0.040
Atm/hpa	1014.45 ± 6.53	1014.48 ± 4.78	1014.95 ± 6.12	1014.99 ± 5.00	1018.22 ± 5.78	2.060	0.142	0.093
BT/°C	36.29 ± 0.46	36.28 ± 0.45	36.30 ± 0.43	36.31 ± 0.47	36.34 ± 0.41	0.143	0.891	0.007

*RT, room temperature; H, humidity; Atm, atmosphere; BT, body temperature; and F, statistic value of variance analysis. η_p_^2^, effect size.*

**FIGURE 3:**
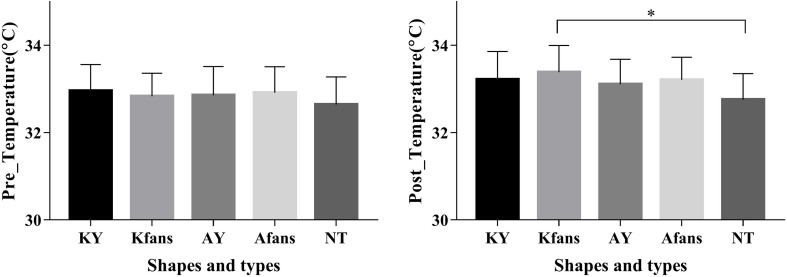
Comparison of skin temperature at post-taping for different taping methods ($\left[\bar{x}\right]$ ± *s*). Note: ^∗^Significant difference between groups, *p* < 0.05.

Significant differences were observed in the short-term effect on skin temperature for different taping methods (*p* = 0.012, *F* = 3.435, and η_p_^2^ = 0.147); *post hoc* test showed a significantly higher skin temperature difference in Kfan taping compared to that of no taping (*p* = 0.026, 95% CI = 0.051–1.206). (Table 4 and Figure 3).

**TABLE 4:** Comparison of skin temperature and self-perceived temperature for different taping methods ($\left[\bar{x}\right]$ ± *s*).

	KY	Kfan	AY	Afan	NT	F value	*P* value	η_p_^2^
Pre_T/°C	32.95 ± 0.60	32.83 ± 0.53	32.86 ± 0.66	32.91 ± 0.59	32.64 ± 0.64	0.958	0.406	0.046
Pos_T/°C	33.21 ± 0.64	33.39 ± 0.61	33.11 ± 0.57	33.21 ± 0.53	32.75 ± 0.60	3.435	0.012*	0.147
Self-Perceived	6.68 ± 1.76	7.23 ± 1.41	6.45 ± 1.47	6.40 ± 1.73	6.16 ± 1.82	2.428	0.055	0.108

Pre_T, pre taping temperature; Post_T, post taping temperature; and Self-Perceived, self-perceived temperature of participants through visual analogue scaling. *Significant difference between groups, *p* < 0.05. F, statistic value of variance analysis, and η_p_^2^, effect size.

Additionally, the self-perceived temperature of Kfan is higher than other taping conditions, although there was no significant difference among different taping methods (*p* = 0.055, *F* = 2.428, and η_p_^2^ = 0.108). (Table 4).

## Discussion

To our knowledge, this was the first study to explore the effect of different taping methods on local skin temperature. Our measurement demonstrated that infrared thermal imaging system exhibit good test-retest agreement in skin temperature of healthy participants. In addition, our results showed that different taping methods could increase the local skin temperature significantly at the waist after taping for 10 min. The skin temperature of the fan-strip of KY was significantly higher than no taping condition, although it remained unclear for the real application.

### Reliability Measurement

This study measured the reliability of the infrared thermal instrument and calculated ICC, SEM, and MD. Compared with ICC which is dimensionless, the SEM is interpretable in the units of variables to evaluate the reliability. The infrared thermal imaging system showed good test-retest agreement based on SEM (0.33°C) and ICC (0.82). As for the MD (0.91°C), this value means that only if the temperature difference between two measurements is greater than or equal to 0.91°C, the change is real at the 95% level (Weir, 2005). However, the MD exceeded the temperature differences that we observed in all the interventions, making the clinical application of KY questionable, despite a respectable SEM. Since our participants were healthy young adults, perhaps larger changes could be observed in the affected population.

### Effect of Taping on Skin Temperature

This findings showed that fan-strip of KY had a significant effect on local skin temperature, which is consistent with previous studies. Slomka et al. (2018) reported that after taping for 4 days, the skin temperature decreased immediately on removal and then increased significantly. This result indicated that KT had a significant effect on local skin temperature changes. Similarly, Windisch et al. (2017) found the skin temperature at the wound site of KT group was higher significantly than control group and concluded that KT contributed to a reduction of swelling after total knee replacement. Conversely, Yang and Lee (2018) indicated that the skin temperature decreased immediately after taping in the ROI. However, they did not describe clearly the ROI of infrared measurement. The ROI temperature measured in our study was only the skin temperature around the tape and did not include skin covered by the tape. The coverage of the tape has an influence on measured skin temperature, which may be the main reason for differences in the results of our study. Skin temperature changes in the area covered by the tape were not reported considering that the tape covering might play a role in insulating the skin of the covered area. Therefore, only the temperature of the skin exposed between the tape and the tape coverage edge were reported in this study. It is suggested determining the emissivity of the tape to better interpret the skin temperature data under the coverage in future research.

The skin is the largest sensory organ of the human body and contains a large number of heat receptors, such as transient receptors and potential receptors (Romanovsky, 2014). Skin vessels activate vasodilation during local warming (Johnson et al., 2014), and animal studies have shown that the intrinsic contractility of lymphatic vessels and lymphatic vessel flow are affected due to temperature regulation (Solari et al., 2017). Our results showed that KT increased significantly local skin temperature in the short-term, with an average increase of 0.56°C in the fan-strip of KY, which created a temporary warming environment to these body parts. Local thermal stimulation has been reported to enhance endothelial cell secretion of nitric oxide (NO) and other substances, and NO participates in the induction of smooth muscle relaxation or vasodilation, thereby increasing local circulation flow (Kellogg et al., 2009).

Previous study showed that when the temperature difference between external and internal (test environment) was less than 5°C, thermal balance could be obtained within a 10-min adaptation period (Marins et al., 2014). Two other studies suggested that the minimum adaptation period should be 15 min (Fernández-Cuevas et al., 2015; Moreira et al., 2017). Therefore, we chose a 15-min adaptation period as the time interval before infrared thermal measurement to exclude the interference of temperature change factors.

### Effect of Taping on Local Circulation

Several studies have confirmed the clinical effect of KT in improving local circulation (Aguilar-Ferrandiz et al., 2014; Vercelli et al., 2017; Windisch et al., 2017; Tantawy et al., 2019). In this study, when participants returned to an upright posture from bending forward, small folds of uniform density were formed in the taping area with KT. Kase et al. (2003) suggested that KT could increase the subcutaneous space, reduce the pressure of pain receptors in tissues, accelerate blood and lymph circulation, and ultimately reduce pain. In subcutaneous tissues, the capillary plexus is located approximately 0.3–0.7 mm below the skin surface, and the capillary network formed extends upward approximately between 0.04 mm and 0.08 mm beneath the skin (Swain and Grant, 1989). Yu et al. (2016) found that KT with natural tension applied to the skin would increase the subcutaneous gap by approximately 0.2 mm. This suggested that the folds produced by KT increased the circulatory space of the subcutaneous capillary network, thereby increasing its blood flow. Therefore, in this study, the application of KT may provide more subcutaneous space and acquire the potential to promote the flow of local microcirculation.

The mechanical load exerted by KT on the skin has been reported to produce extensive heterogeneous deformation (Alexander et al., 2008; Pamuk and Yucesoy, 2015), which forms a certain shear stress within the subcutaneous tissues and the extracellular matrix and between the continuity of connective tissue and muscle fibers (Berthier and Blaineau, 1997; Yucesoy et al., 2003; Huijing, 2009), and allowing the transmission of force between the myofascial membranes (Yucesoy and Huijing, 2007; Yucesoy, 2010). The increased shear stress in the blood vessel wall may increase mechanical stimulation of blood vessels, thereby inducing the secretion of NO, which has a strong vasodilator effect and an important role in increasing blood flow and promoting local microcirculation (Buga et al., 1991; Paniagua et al., 2001). It was reported that the increase in local blood flow has been reported to promote an increase in skin temperature (Tauchmannova et al., 1993; Ng, 2009; Gomes Moreira et al., 2017). That may be the underlying reason for the increase of skin temperature observed in our study.

### Effect of Different Taping Methods on Skin Temperature

We found there was a short-term skin temperature increase using the fan-strip KT method, which was higher significantly than that of no taping. Yu et al. (2016) showed that the subcutaneous gap generated of KT was related to a recoiling force, and this recoiling force was not the same when using different methods of KT application. In this study, due to the proportional distribution of KT tension, we selected the strip of Y and fan taping which were used commonly in clinic. The fan-strip of KT skin temperature was higher significantly than the no taping in the short-term, which is consistent with Yu et al. (2016) findings showing that the recoiling force of the corresponding shape of the taping is approximately proportional to its area and distribution. Fan-strip of KT generates wider range of skin folds (subcutaneous gaps) and elastic recoil force on the skin. This may be one of the main reasons for temperature change in the skin near fan-strip KT.

Vercelli et al. (2017) applied the fan-strip of KT as a form of treatment for postoperative swelling and reported that KT did not change significantly the color intensity of the central area of taping but showed significant therapeutic effect at the edge of the KT, which suggested that the efficacy of KT may related to the formation of a pressure gradient between KT and adjacent area. Pamuk and Yucesoy (2015) used magnetic resonance imaging (MRI) analysis to evaluate local tissue deformation that occurred immediately after KT application and considered that KT promoted lymphatic drainage not only through lifting the skin but also through compressing the skin, which may depend on the pressure gradient in the tissue. The pressure gradient of KT generated from the skin is only distributed at the peripheral edges, and the pressure gradient of fan-strip KT was several multiples of no taping and Y-strip KT. Therefore, fan strip of KT was estimated theoretically to promote local blood flow more quickly and effectively, thereby changing the local skin temperature.

### Effects of Two Different Fan-Strip Materials on Skin Temperature

Previous studies have shown that the skin surface temperature is closely related to subcutaneous perfusion and tissue metabolism and that skin infrared thermal radiation may reflect an increase or a decrease in local skin perfusion flow rates (Slomka et al., 2018). In this study, after using two different taping materials, we found the temperature difference at the waist in Kfan was significantly higher than that of no taping, but this was not observed with Afan. This phenomenon may have been due to the viscoelastic properties and thickness which similar to human skin. KT has good elastic properties and does not produce pressure on the deep tissues to restrict their movement (Hosp et al., 2018). Compared to KT, AT has poor viscoelasticity, and a weak pulling effect on skin; therefore, the subcutaneous gap and the thermal effect are less than that of KT.

In addition, we found that there was no significant difference for self-perceived temperature after taping for 10 min. This result indicated that the self-perceived temperature was not related to the materials and taping methods. However, there was an increase of self-perceived temperature, this may be due to the placebo effect which affected psychological expectations of participants.

### Limitations

However, the limitations must be considered when interpreting the results. Firstly, we only observed temperature fluctuations at the waist after taping. As a core region of the human body, the temperature of the waist is relatively high with little fluctuation, while the temperature of the distal extremities may be more susceptible to external intervention (Hermanns et al., 2018). Secondly, it has been reported that skin temperature distribution is influenced by the subcutaneous fat of each body segment. (Neves et al., 2015; Salamunes et al., 2017), the lack of fatness measurement was a further limitation, which is important for skin temperature distribution. Additionally, all participants were healthy, whereas symptomatic patients may present with abnormal temperature distribution which may have more obvious response for taping. We neglected the reliability analysis in different taping interventions, which may affect the accuracy of the experimental results. Lastly, this study only observed the short-term effects of different taping methods on local skin temperature under static conditions, and dynamic and long-term effect were needed to be verified furtherly.

### Future Research

Due to taping was used commonly in dynamic conditions during real complex environments, future research needs to involve the effects of different taping methods KT on skin temperature under a dynamic condition (e.g., during exercise; Trecroci et al., 2019). In addition, we also need to develop blinded, randomized controlled trials with greater sample size, extend the taping time, compare more types tapes such as spatial, or drift taping in order to explore time-effect relationship of different taping methods on the skin temperature combined with physiological mechanisms at the level of cell and blood.

## Conclusion

This study indicated that the fan-strip of KY increased significantly the skin temperature of the waist after taping for 10 min among healthy adults. The application of KT may modify the skin temperature of the human body and promote local microcirculation, although it remained unclear for the real application.

## Data Availability Statement

The datasets generated for this study are available on request to the corresponding author.

## Ethics Statement

The studies involving human participants were reviewed and approved by Ethics Committee of Shanghai University of Sport, ID 2018071. The patients/participants provided their written informed consent to participate in this study.

## Author Contributions

KL contributed to recruit the subjects, collect the data and writing the manuscript. ZD, LC, ZW, and SZhu contributed to recruit the subjects, and collect the data. QQ, WC, and SZha undertook statistical analysis. BY conceived of the study and interpreted the results.

## Conflict of Interest

The authors declare that the research was conducted in the absence of any commercial or financial relationships that could be construed as a potential conflict of interest.
