# Supplementary material for: Short-Term Effect of Different Taping Methods on Local Skin Temperature in Healthy Adults
Source: Front Physiol. 2020 May 20;11:488. doi: 10.3389/fphys.2020.00488 (PMC7251151; doi:10.3389/fphys.2020.00488)
Supplement: Supplementary file 1 [file Data_Sheet_1.PDF]

SUPPLEMENTARY MATERIAL 1

Two balanced Latin Square was constructed. In the first Latin Square, the first row follows the formula 1, 2, n, 3, n-1, 4, n-2…, where n is the serial number of taping conditions. For subsequent rows, add one to the value corresponding to the previous line, returning to 1 after n. The second Latin Square is a mirror image of the first Latin Square. This incomplete counterbalanced randomization method is applied for repeated measures design with many conditions. It could ensure that every single condition follows every other condition once, avoiding any carryover effects during the statistical analysis.

S1 Two balanced Latin Square

**Participant**	**Order of taping conditions**				
Participant 1	1	2	5	3	4
Participant 2	2	3	1	4	5
Participant 3	3	4	2	5	1
Participant 4	4	5	3	1	2
Participant 5	5	1	4	2	3
Participant 6	4	3	5	2	1
Participant 7	5	4	1	3	2
Participant 8	1	5	2	4	3
Participant 9	2	1	3	5	4
Participant 10	3	2	4	1	5
Participant 11-20	Same with participant 1-10				
Participant 21	1	2	5	3	4

1= Y-strip of kinesio taping; 2= fan-strip of kinesio taping; 3= Y-strip of athletic taping; 4= fan-strip of athletic taping; 5=No taping.
