# Supplementary material for: Short-Term Effect of Different Taping Methods on Local Skin Temperature in Healthy Adults
Source: Front Physiol. 2020 May 20;11:488. doi: 10.3389/fphys.2020.00488 (PMC7251151; doi:10.3389/fphys.2020.00488)
Supplement: Supplementary file 2 [file Data_Sheet_2.PDF]

SUPPLEMENTARY MATERIAL 2

The system specification and camera specification of Spectrum 9000 mb-500 FT digital infrared thermal imaging system (United Integrated Services Co, Ltd, New Taipei, Taiwan, China)

Sensor types	UN-cooled Microbolometer
Infrared detection band	7-14μm
Thermal image resolution	320×240 pixels
Image resolution	640×480 pixels
Reading accuracy	＜0.3℃ Authenticated by the notary organ compared with standard temperature source
Reading stability	＜0.3℃ Authenticated by the notary organ compared with standard temperature source
System accuracy	0.1℃
scanning frequency	60Hz
Minimum analytical temperature difference	0.05℃~0.08℃
camera angle	20°（level）×15°（vertical）
Temperature measurement range	10℃~40℃
Photography distance	60cm~ infinity
Operating temperature	18℃~28℃
Lens type	F/# 1,50mm
Spatial resolution	≤1.0mrad
Ambient temperature feedback device	0.1℃ Automatic correction of ambient temperature per second
